# A real-life observational study of the effectiveness of FACT in a Dutch mental health region

**DOI:** 10.1186/1471-244X-8-93

**Published:** 2008-12-04

**Authors:** Marjan Drukker, Myrte Maarschalkerweerd, Maarten Bak, Ger Driessen, Joost à Campo, Arthur de Bie, Giovanni Poddighe, Jim van Os, Philippe Delespaul

**Affiliations:** 1 Department of Psychiatry and Neuropsychology, South Limburg Mental Health Research and Teaching Network, EURON, Maastricht University, PO Box 616, (location Vijverdal) 6200 MD Maastricht, the Netherlands; 2 Integrated Care and f-ACT (Psycope), Mondriaan, South Limburg, the Netherlands; 3 Prins Claus Centrum (Mental Health Centre), p.o. box 5500, 6130 MB Sittard, the Netherlands; 4 Division of Psychological Medicine, Institute of Psychiatry, De Crespigny Park, Denmark Hill, London, UK

## Abstract

**Background:**

ACT is an effective community treatment but causes discontinuity of care between acutely ill and currently stable patient groups. The Dutch variant of ACT, FACT, combines both intensive ACT treatment and care for patients requiring less intensive care at one time point yet likely to need ACT in the future. It may be hypothesised that this case mix is not beneficial for patients requiring intensive care, as other patient groups may "dilute" care provision. The effectiveness of FACT was compared with standard care, with a particular focus on possible moderating effects of patient characteristics within the case mix in FACT.

**Methods:**

In 2002, three FACT teams were implemented in a Dutch region in which a cumulative routine outcome measurement system was in place. Patients receiving FACT were compared with patients receiving standard treatment, matched on "baseline" symptom severity and age, using propensity score matching. Outcome was the probability of being in symptomatic remission of psychotic symptoms.

**Results:**

The probability of symptomatic remission was higher for SMI patients receiving FACT than for controls receiving standard treatment, but only when there was an unmet need for care with respect to psychotic symptoms (OR = 6.70, p = 0.002; 95% CI = 1.97 – 22.7).

**Conclusion:**

Compared to standard care, FACT was more rather than less effective, but only when a need for care with respect to psychotic symptoms is present. This suggests that there is no adverse effect of using broader patient mixes in providing continuity of care for all patients with severe mental illness in a defined geographical area.

## Background

The Dutch schizophrenia guidelines recommend assertive community treatment (ACT) as the primary service provision for people who have a severe mental illness (SMI) and are reluctant to work with mental health care services [1]. Earlier studies and the literature review underlying this recommendation have shown that ACT is superior to standard treatments in a wide range of clinical and service outcomes [1-6], but these studies originate primarily from the US.

In The Netherlands, a Dutch variant of ACT was developed: 'function-ACT' (FACT) [7]. FACT teams are delivering service for the total group of SMI patients in a region, where ACT only covers the 20% most severely ill subgroup. In FACT teams, care intensity can be upgraded when needed. Therefore, FACT combines two approaches within one multidisciplinary recovery-oriented team: (a) individual case management and home visits for extensive care SMI-patients who are mostly stable; (b) shared caseload with intensive full ACT approach for patients in need for intensive care. The latter group receives care according to ACT directives including daily review in staff meetings. When, over time, the care needs of these patients change, they remain in the same multidisciplinary team (continuity of care). Members of FACT teams conduct a style of assertive outreach by paying both announced and unannounced home visits to both groups of patients, and by using supportive legal measures when needed [8]. Compared to ACT, FACT is a more versatile and comprehensive care system with continuity of care as an important component. The FACT teams are in charge during both outpatient episodes and admission and decide on discharge [7]. Thus, FACT teams serve a diverse population of SMI patients with various levels of need for care. On the other hand, "classic" ACT serves only those SMI patients who are in crisis or have the highest needs for care. As intensive care patients need more guidance than more stable patients do, the average frequency of contacts in a FACT team is lower and the number of patients per team is higher than in an ACT team. Although FACT was developed for the Dutch mental health care system, it has been noted that its features may also be applicable in other countries, such as the U.S. [9].

Only a few European studies have assessed effectiveness of ACT or FACT. In Europe, effects of ACT are not as positive as suggested in early U.S. work. Some European studies have found a beneficial effect for ACT in length and number of hospitalizations [10-14], continuity of care [11,15-17], psychopathology [18-21], social functioning [19] or housing stability ([14]. However, these beneficial effects are not always significantly superior to the effect of other community-based treatments. In The Netherlands, Dekker and colleagues [12] conducted one of the few RCT studies of the effectiveness of Dutch ACT teams. They compared SMI patients receiving either ACT or standard outpatient treatment on number and length of hospital admissions, general functioning, psychopathology and social behaviour. A large reduction in number of bed days was seen for the experimental condition in comparison to the control group after two years of ACT, but no differences between ACT and the control treatment were found for any of the other outcome variables [12]. In another Dutch RCT study of the effectiveness of ACT, the primary outcome was loss to follow-up over a period of two years [17]. This study showed that FACT was significantly better than standard care in maintaining contact with patients. However, the decrease in drop-out rates of FACT patients was not reflected in any of the clinical outcome variables, such as psychopathology, social functioning and quality of life. In contrast to these two Dutch studies, Bak and colleagues [18] did find an effect of FACT on a clinical outcome [18]. In a mirror-image study, they compared the number of patients with a psychotic disorder who were making the transition to symptomatic remission before and after the introduction of FACT in Maastricht, and found that the probability of going into symptomatic remission increased by 15% (Number Needed to Treat) after the introduction of FACT. After controlling for possible confounders, this risk difference decreased only slightly – namely to 12% – although it was no longer statistically significant.

A reason for the less positive results in Europe when compared with the U.S. could be that in studies where comparative (standard) mental health practice is poor, effect sizes of ACT are higher [10,22]. When ACT was developed in the U.S., patients were discharged into a community with almost no mental health care [23]. Standard treatment was either expensive hospital care for a few or nothing at all for most patients. In Europe, a reasonably well-developed community-based mental health service exists as the 'standard' treatment. In addition, in FACT the focus of treatment is the community rather than the hospital, not only for the 80% less intensive SMI patients but for all SMI patients. The aim is that the more severely ill SMI patients also benefit from social inclusion in the community [7]. In addition, outpatient SMI patients can access resources needed for recovery and rehabilitation that were often only available for hospital patients.

Currently, more than 60 FACT (and 25 ACT) teams are in operation in the Netherlands (population 16.4 million); an increase to 200 FACT teams is expected [7]. As more FACT teams are being set up in The Netherlands it is important to assess whether FACT is more effective than other community-based treatments in targeting SMI patients. As stated above, FACT teams serve a heterogeneous population. Because of this heterogeneity, it can be questioned whether effectiveness of FACT is the same for all FACT-patients. As far as we know, it has never been studied whether FACT is beneficial for all patients receiving it. The FACT-specific case mix of patients needing either intensive or extensive care may "dilute" care and create poorer outcome for patients with the most treatment needs. Therefore, effect modification between FACT and need for care on symptoms and self-care as well as between FACT and functioning may be hypothesized. In addition, SMI patients with comorbid alcohol addiction (dual disorder patients) are a more severe subgroup within the FACT population, possibly more difficult to treat. For example, it has been shown that these patients more often use crisis services and are hospitalised and involuntarily admitted more frequently [24,25].

The present observational study was set up to assess the effectiveness of FACT in the south of the Netherlands. Although the design of RCT studies is sound, the research requirements can limit the external validity and generalizability to different settings. The effectiveness of psychosocial interventions such as FACT depends on environmental factors – such as time per patient, type of mental health organization and compliance to the treatment protocol – much more than pharmacological compounds do [22,26,27]. While RCT studies assess treatment effectiveness in theory, effectiveness in practice should additionally be studied in observational studies that do not manipulate the environment. Therefore, an observational study was chosen over an RCT.

The present study was an extension of the study of Bak and colleagues [18]. Instead of a pre-post mirror-image study design, which may be confounded by changing circumstances over time, it compared the probability of being in symptomatic remission for patients receiving FACT versus similarly affected patients receiving standard treatment. It was hypothesized that patients receiving FACT have a higher probability of being in symptomatic remission over a period of time than patients receiving standard treatment. In addition, given the FACT-specific case mix of patients we examined to what degree effectiveness may be differential depending on factors determining the intensiveness of need for care, including: functioning/self-care treatment needs, substance use needs (as indicators for dual disorder), psychotic symptom treatment needs and level of chronicity (new in care, 2–3 years in care, chronic).

## Methods

Since 2002, three FACT teams have been set up in Maastricht and surrounding areas in the province of Limburg, which is in the south of the Netherlands. These teams serve all SMI patients living independently, in sheltered accommodation or in an open psychiatric hospital ward [8]. Standard treatment of patients with SMI – the control condition – includes inpatient treatment, sheltered residential treatment and community treatment with broker-type case management.

### PCR and CNCR

The data for the present study were obtained from a merged database consisting of the Psychiatric Case Register (PCR) and the Cumulative Needs for Care Register (CNCR). The PCR registers the consumption of mental health care of psychiatric patients in South Limburg (population 660 000). Data for the PCR have been provided by all regional mental health facilities for all patients since 1981 [28]. The CNCR monitors the need for care of patients with SMI in South Limburg. The data for the CNCR have been collected in Maastricht since 1998 and in the other regions of South Limburg since 2004. Data are cumulatively and routinely collected in clinical practice with intervals of 1 to 2 years and with every major change in treatment or setting. There is no specific baseline in the flow of the illness history and the number of follow-up measurements differs per person as this is a naturalistic study [29]. For the present analyses, the CNCR and PCR were anonymously matched using a case identification code with an encryption algorithm and the date of the CNCR interview [18] and values of the first assessment after 2002 were used as a proxy for baseline values.

### CNCR interview

A CNCR interview [29] provides information on (i) demographic variables, (ii) current level of psychopathology, (iii) level of general functioning, (iv) needs for care, v) quality of life (5 items) and vi) quality of care (1 item).

Psychopathology was assessed using the Brief Psychiatric Rating Scale (BPRS). The BPRS [30] is a semi-structured interview assessing the presence and severity of various symptoms in the two weeks preceding the interview. The BPRS items are scored on a 7-point Likert scale (1 = not present; 7 = most severe). Ratings are based upon clinical observations of symptoms and patients' verbal report of symptoms. In order to standardize the ratings, anchor points and probe questions are described for each item [29,30]. Factor analyses of the BPRS have revealed the existence of four underlying constructs ('BPRS scales'): negative symptoms, positive symptoms, manic excitement and depression/anxiety [31]. The BPRS items blunted affect, motor retardation, emotional withdrawal and self-neglect loaded on negative symptoms; bizarre behaviour, unusual thought content, disorientation, hallucinations and suspiciousness loaded on positive symptoms; motor hyperactivity, elevated mood, excitement, distractibility, hostility and grandiosity loaded on manic excitement; and depression, anxiety, suicidal ideation and guilt loaded on depression/anxiety.

Need for care was assessed using the Camberwell Assessment of Need (CAN) [32]. Baseline values of a few individual items were used in the present paper (see statistical analysis) because the sum score lacks validity and, therefore, is likely of less value [33]. The CAN includes 22 items (e.g. daytime activities, psychotic symptoms). All CAN items can be scored 0 (no problem), 1 (there was a problem, but the problem is met), 2 (unmet need) with a reference period including the last 3 months [8]. In the CNCR, information from clinician and patient is combined using *a priori *decision rules to maximise the clinical relevance of the rating.

Information on the global functioning of patients was obtained with the extended Global Assessment of Functioning (GAF) scale [34]. This tool consists of a psychopathology scale (GAF-p, sample range 1–95), rating the global symptom severity of patients, and an impairment scale (GAF-i range 1–95), rating their level of impairment in psychosocial functioning.

### Outcome measure

The primary outcome measure was symptomatic remission of psychotic disorder. Symptomatic remission is defined as a symptom intensity level within the normal general population range, reflecting negligible influence on an individual's functioning [35]. For the objective measurement of symptomatic remission the following 7 BPRS items are included [35,36]: 1) grandiosity, 2) suspiciousness, 3) unusual thought content, 4) hallucinatory behaviour, 5) conceptual disorganization, 6) mannerism/posturing and 7) blunted affect [35,36]. These BPRS items are diagnostically specific to psychotic disorders. They reflect both the three dimensions of psychopathology underlying psychotic disorders (psychoticism, disorganization, negative symptoms) and the five acute phase criteria of schizophrenia in the DSM IV (delusions, hallucinations, disorganization, disorganized or catatonic behaviour, negative symptoms) [35,36]. In order to be classified as being in symptomatic remission, patients had to score 3 or less on all 7 BPRS items [35,36].

### Subjects and matching

Data of SMI patients collected between April 2002 (the start of FACT in the Maastricht region) and August 2007 were included in the matching procedure. Of the matched patients, data up to December 2007 were included in the multilevel analyses (see below). Patients were classified as SMI patients if they fit the diagnosis of schizophrenia or psychotic disorder (DSM-IV 295, 297 or 298), or had a minimal score of 15 on the positive symptom scale of the BPRS on any of the assessments. Additionally, patients with a low level of functioning (below 45 on at least one of the GAF scales) and a minimum of 2 needs in the domains of accommodation, welfare benefits, alcohol and drugs, were classified as SMI patients [33]. These four domains of needs were *a priori *selected from the 22 CAN domains. Because services for alcohol and drug addiction did not take part in the data collection, patients with substance abuse disorder with no further psychiatric disorders were not included. As the definition was to a degree arbitrary and an absolute cut-off may be too strict, the group of patients scoring below 45 on one of the two GAF scales and a need in 1 of the 4 above-mentioned CAN domains was also included in the analyses as moderately mentally ill (MMI) rather than SMI patients. All patients not meeting the above criteria for MMI and SMI were excluded from the analyses, even if they were treated by a FACT-team.

Next, all patients ever identified by either the PCR or the CNCR as receiving FACT were coded as FACT patients. FACT patients are a more severely ill subgroup of all patients in the CNCR database. Consequently, patients coded as receiving FACT were matched with controls, using propensity score nearest neighbour-matching (one on one) with replacement. Propensity scores were based on age category (18–30, 31–65, 66+), the two GAF-scales and the four BPRS sum scores (all continuous variables). The FACT group included Maastricht patients only, while Maastricht patients were excluded when selecting matched controls, because all Maastricht patients who were eligible for FACT were already served by one of the FACT teams. Before matching, FACT patients differed significantly from all non-FACT patients with respect to age (mean age FACT-patients 38 years, others 43 years) and GAF (mean symptoms FACT-patients 52.9, others 50.7). Positive symptoms and negative symptoms were higher in the FACT group and depression/anxiety was lower, but differences were not statistically significant. After the matching, matching variables were more balanced than before, although there was still a small difference in age (mean 37 years vs 39 years, p = 0.08).

### Statistical analysis

All analyses were performed using the statistical program Stata 10 [37]. Since the data consisted of multiple measurements per patient and the patients were matched the observations were not independent. As a result, standard logistic regression techniques could not be used for statistical analysis of the data. The data with measurements (level 1) clustered in subjects (level 2) clustered in matched groups (level 3) were, therefore, subjected to multilevel logistic regression analysis, which is ideally suited for analysis of this type of data [38]. The xtmelogit command is the logistic regression variant of the Stata xtmixed command with 2 or more levels. The group level was added to control for clustering in the data introduced by the matching. The dependent variable was symptomatic remission (yes/no) a variable at measurement level, while the independent variable was FACT (yes/no). Age, symptom severity at baseline (four BPRS continuous variables), functioning at baseline (two GAF scales, continuous) and gender were included in the models as independent variables.

In addition, interaction terms between FACT and a set of baseline variables as outlined earlier were included in the model: GAF symptoms and handicap, level of chronicity, CAN alcohol, CAN drugs, CAN psychotic symptoms and CAN self care. When any of these interaction terms was statistically significant (alpha was set at 0.05) the Stata Lincom procedure was used to calculate odds ratios of FACT and remission for all strata of the interaction variables.

## Results

Two-hundred forty patients receiving FACT were matched with two-hundred controls. FACT-patients were on average 2.7 times (range 1–8) and non FACT-patients 2.3 times assessed (range 1–5). Table 1 presents the characteristics and the remission status of the patients.

**Table 1:** Demographic and diagnostic characteristics of the non-FACT and FACT patients

	Non-FACT patients (n = 200)	FACT patients (n = 240)
**Remission at assessment **(%)	123 (61.8)^1^	153 (63.8%)^1^
**Men **(%)	125 (62.5)	145 (61.4)

**Ever SMI**		
SMI (%)	141 (70.5)	200 (83.7)
MMI (%)	59 (29.5)	39 (16.3)

**Baseline:**		
**Remission **(%)	108 (54.3)	118 (49.2)

**Level of chronicity**		
New	22 (13.4)	19 (11.7)
2–3 years	14 (8.5)	12 (7.4)
Chronic	128 (78.1)	131 (80.9)

**Need for care psychotic symptoms**		
No	75 (38.5)	62 (26.7)
Met	79 (40.5)	111 (47.8)
Unmet	41 (21.0)	59 (25.4)

**Need for care alcohol**		
No	146 (76.0)	189 (80.1)
Met	27 (14.0)	25 (10.6)
Unmet	19 (9.9)	22 (9.3)

**Age**		
Mean (SD)	39.3 (12.2)	37.3 (11.8)
Range	19–77	18–81

**GAF Psychopathology**		
Mean (SD)	53.8 (15.8)	53.3 (15.9)
Range	5–90	15–95

**GAF Impairment**		
Mean (SD)	51.6 (13.7)	50.5 (15.0)
Range	20–81	18–95

**BPRS Depression/anxiety**		
Mean (SD)	9.9 (4.5)	9.6 (4.6)
Range	4–23	4–23

**BPRS positive symptoms**		
Mean (SD)	9.5 (5.1)	9.6 (4.4)
Range	5–33	5–26

**BPRS negative symptoms**		
Mean (SD)	6.7 (3.1)	6.6 (3.1)
Range	4–25	4–21

**BPRS manic excitement**		
Mean (SD)	9.7 (4.3)	9.4 (3.8)
Range	6–36	6–23

^1 ^Note that remission is assessed at measurement-level and a patient can be in remission at one moment and not in remission at another and, therefore, the numbers add up to more than the total number of patients.

Analyses showed statistically significant positive interaction between FACT and baseline need for care with respect to psychotic symptoms (χ^2 ^= 12.62, df = 2, p = 0.002) and a significant negative interaction between FACT and baseline need for care with respect to alcohol (χ^2 ^= 6.74, df = 2, p = 0.03). Thus, FACT patients with an unmet need in psychotic symptoms were more often in remission at follow-up than non-FACT patients (OR = 6.70, p = 0.002; 95% CI = 1.97 – 22.7, table 2). If additionally the interaction with need for care with respect to alcohol use was taken into account, the odds increased further: remission was more than 8 times more likely in those with unmet need for psychotic symptoms and *absence *of a need with respect to alcohol use (OR = 8.52).

**Table 2:** Odds ratio's (95% confidence intervals) of FACT when studying remission, depending on baseline need for care on psychotic symptoms

	need for care at baseline: psychotic symptoms		
	no	met	unmet

	0.35* (0.14 – 0.87) n = 137	1.07 (0.55 – 2.05) n = 190	6.70** (1.97 – 22.7) n = 100

need for care at baseline: alcohol			

no	0.54 (0.21 – 1.37) n = 100	1.43 (0.72 – 2.84) n = 156	8.52** (2.43 – 29.8) n = 75

met	0.16* (0.03 – 0.80) n = 19	0.42 (0.09 – 1.97) n = 21	2.50 (0.43 – 14.6) n = 10

unmet	0.07** (0.01 – 0.43) n = 17	0.19 (0.03 – 1.04) n = 13	1.12 (0.15 – 8.31) n = 11

* p < 0.05

** p < 0.01

## Discussion

Results of the present study showed that, within the subgroup of patients with an unmet need for care with respect to psychotic symptoms at baseline, patients receiving FACT were more likely to be in remission than were patients receiving standard treatment. This suggests that FACT makes a difference in patients for whom either the patient or the clinician recognises an unmet need in this area. However, a coexisting alcohol problem precluded any associations with FACT.

A first explanation for only finding an association between FACT and remission in patients scoring an unmet need on psychotic symptoms could be that these patients may be the most severely ill. The conclusion that FACT is more effective than standard care for the high care patient subgroup and should be restricted to these patients, could be a consequence of a "regression to the mean" effect. However, if severity was the only explanation, we would have expected an interaction between severity of symptoms and FACT. *Post hoc*, an interaction term between FACT and BPRS positive symptoms was added to the regression model. This interaction term was statistically imprecise by conventional alpha, while the interaction between FACT and CAN psychotic symptoms remained. In addition, patients with an unmet need in psychotic symptoms are not per definition the most severely ill [see [29]], although it is likely that severely ill psychotic patients will have a need for care on that item. This is verified by the correlation between unmet need with respect to psychotic symptoms and BPRS positive symptoms, which is relatively high, but not perfect (r^2 ^= 0.6) and higher than the other three BPRS sum scores (r^2 ^between 0.17 and 0.21, all correlations p < 0.001).

There are several other explanations for finding an association in patients with an unmet need with respect to psychotic symptoms, only. First, in standard care, a patient with a need for care with respect to psychotic symptoms is treated by a single professional, often a psychiatrist who provides medication to manage symptom exacerbation. In FACT, a multidisciplinary team provides treatment for all SMI-patients rather than only for those SMI patients who need intensive care. This team includes a psychiatrist, psychologists, addiction workers, case managers and vocational rehabilitation workers using Individual Placement and Support (IPS). Professionals from the multidisciplinary team start treatment in their area of expertise, in parallel to the psychiatrist who reduces psychotic symptoms. Because of the continuity-of-care principle these interventions are continued when crises are managed.

Second, over the years, some ACT related principles became 'good practice' references and were included in standard care. This could be a reason why no extra beneficial effects could be shown in the group of patients without a need for care with respect to psychotic symptoms.

The present study also showed that a need for care in the area of alcohol precluded the beneficial effects of FACT. Patients with alcohol dependence do not seem to benefit from FACT. This is not in line with a previous study showing that high fidelity to ACT (including dual disorder treatment) is of particular importance for substance abuse outcomes in dual disorder patients ([39]. In Maastricht the fidelity to ACT guidelines is acceptable (see below), but addiction 'specialists' were not yet available during the study assessment period. SMI patients with comorbid alcohol addition may be more difficult to treat than other SMI patients, resulting in increased use of crisis services and higher percentages of self-harm [24]. In a London study, dual disorder patients were more often hospitalised or involuntary admitted and the authors speculated that these patients might benefit from specific interventions, such as a specialized FACT team for dual disorder patients [25]. Fidelity guidelines for FACT-teams in the Netherlands do list Integrated Dual Diagnosis Treatment as a core feature and FACT-teams in other Dutch regions did integrate these. Currently Integrated Dual Diagnosis Treatment is progressively implemented in the Maastricht FACT teams. It is expected that this would improve outcomes for dual disorder patients.

Other European and recent US studies showed less impressive effect sizes than the present study [12,17,19-21]. The focus of FACT on early detection of symptom exacerbation to prevent more serious symptomatology, may explain the difference [7]. Another reason could be fidelity, because lower fidelity has been associated with a lower effectiveness [6,14,39]. Preliminary results using the DACTS – a scale measuring ACT fidelity [40] showed that two Maastricht FACT teams had acceptable scores on fidelity for ACT, while a third team had a moderate score (personal communication Van Vugt). A *post-hoc *analysis restricted to patients of the two teams with acceptable fidelity and their matched controls showed associations between FACT and symptomatic remission that were similar to the original analysis (in patients with an unmet need on psychotic symptoms: OR = 7.06; p = 0.004; 95% confidence interval = 1.8 – 27.1, n = 88; in patients with both an unmet need on psychotic symptoms and no need with respect to alcohol: OR = 8.39, p = 0.003, 95% CI = 2.11 – 33.4, n = 67). Recently, a Dutch expert group embedded in the Centre for Certification ACT & FACT  evaluated working ingredients and formulated fidelity criteria to evaluate FACT rather than ACT. This resulted in the development of a FACT Fidelity Scale (FACTs) and currently field tests are being conducted.

As a result of the integrated patient mix in FACT, it was impossible to isolate the cases within the FACT-teams that were effectively receiving ACT in the present study. Therefore, this is one of the first FACT outcome studies. Further research is needed to replicate the present results. These future studies should include interaction terms and they should assess and control for the fidelity of the FACT teams. It is also important that future studies clearly describe the ingredients of FACT as well as standard treatment, so that effective elements can be identified.

In sum, results of the present study support the recommendation of the Dutch schizophrenia guidelines, which recommend ACT as the primary treatment for people with SMI [1]. Within the Dutch mental health care system this can be operationalised by starting FACT teams. The addition of an addiction specialist to the teams may improve benefits for patients with a coexisting alcohol problem. Although only patients with an unmet need on psychotic symptoms seemed to benefit from FACT, we feel that all patients with or without an unmet need in this area should remain in FACT, because continuity of care is an important feature, probably contributory to the effectiveness of FACT.

### Methodological issues

The strength of the present study is the unique data collection, as real-life observational data were obtained longitudinally within the treatment process and interviews were conducted by different interviewers in different settings. This might increase the generalizability of the results. However, it can also threaten reliability. To minimize this threat, a manual for the assessment of the interviews was developed, interviewers were trained on a regular basis (booster sessions) and new mental health care professionals received supervision in the scoring of CNCR interviews. Another possible threat to the reliability of the data collection is that the interviewers were the mental health care workers involved in the treatment of the subjects and that they were not blinded for the treatment condition. This is, however, hypothesized not to pose a big threat to reliability as the assessment of CNCR interviews were in place years before the introduction of the FACT teams and were never positioned in an evaluation of FACT.

A second important strength of the present study is its outcome measure. Symptomatic remission has been shown to be a clinically meaningful concept and is a feasible outcome measure [36]. The outcome measures most commonly used in studies of the effectiveness of ACT/FACT are the number and/or length of hospital admissions ([10,17,19,27,41], but several studies have shown that these outcomes correlate more with the number of available beds than with the service that is being delivered [27,41]. Consequently, the present study used a clinical outcome measure. However, the definition of full remission, rather than symptomatic remission, is not only based on 7 BPRS items, but also includes a time criterion: symptom levels should remain low for at least 6 months. This time criterion could not be incorporated because only a few patients in this sub sample were measured more than twice and observations were mostly more than 1 year apart. Bak and colleagues showed in a sensitivity analysis that when remission was defined as scoring low on the 7 BPRS items at two successive moments, results were even stronger than the original results [18]. Therefore, it is likely that including the time criterion would have shown similar or even stronger results in favour of FACT, rather than weaker effect sizes.

In addition, this is a naturalistic study of regular clinical practice and the CNCR started in 2004 in the control region, while the first FACT team was established in 2002. Therefore, data from FACT patients and their matched controls could be a maximum of two years apart and the first included assessment occurred after FACT introduction. Furthermore, some best-match controls contributed only one assessment. Analyses may have been methodologically more sound if changes since baseline were analysed, by controlling for remission at baseline and excluding the baseline assessment. Two other ACT studies studied changes in symptom severity [12,17] and these studies did not show any effect. However, according to Andreasen and colleagues [35] 'the real-world interpretability of change scores as a primary outcome is limited because of the variability of baseline symptom severity across intervention trials'.

The current data differ from those used by Bak and colleagues [18] because information from the PCR as well as the CNCR was used to identify which patient actually received FACT. However, the data of the merged PCR and CNCR databases did not always concur with respect to service delivery: some patients were identified as being a FACT patient in one database but not in the other. Therefore, a person was assigned to the FACT group if he/she was classified as receiving FACT at least once in either of the databases. One reason for these differences is that PCR data from the second half of 2006 and from 2007 were not available yet. Although it is possible that some FACT patients were missed, none of these FACT patients were included in the control group, because these were obtained from an adjacent region.

Propensity score matching is a relatively new method of matching which can be used in observational data where treated subjects differ systematically from controls [42]. However, this type of matching can be performed in several different ways and it is unclear which option is the best. Therefore, we performed nearest neighbour matching both with and without replacement [43]. Fortunately, results of the different analyses were rather similar. In addition, an extra level was included in the multilevel analyses to control for the matching [42].

The present study has some other limitations. First, non-FACT patients originated from an adjacent region in which FACT had not yet been introduced. However, the South Limburg patient population is similar in the two sub regions and stable over time. Therefore, invalidation of the results is negligible.

Second, matching, using the first measurement after the start of FACT could have obscured the effect of FACT (FACT patients and controls are similar because the patients outcomes already improved). The current analysis strategy is the best possible to obtain ecologically valid results and the reported association between FACT and remission cannot be a result of the matching using first measurement data.

Third, the CNCR protocol prescribes that CNCR interviews should be carried out at intake, as part of the yearly evaluation, and at every mutation in the patient's care plan. However, due to pragmatic and logistic reasons, professional carers do not always comply exactly. It is possible that members of FACT-teams are better compliers than professional carers in standard treatment, because the CNCR interview is implemented within FACT. In addition, FACT aims to serve patients throughout their course of illness, also offering low-profile supportive care while standard care patients with the same severity of the symptoms are transferred to a less intensive type of care. Therefore, patients in remission may be interviewed more often when served by a FACT-team and this could explain part of the effect reported in the present paper. However, reminders are routinely sent to all interviewers who do not turn in the yearly reassessment and by matching CNCR data with PCR data it is in future also possible to send reminders with every mutation in treatment. Unfortunately, this feedback procedure has a delay of some months. Furthermore, the management in the control region enforces the professional carers to fill in the CNCR forms. Finally, the helpdesk in the control region is very strict in sending reminders and professional carers are also personally approached if necessary. Therefore, we feel that the difference in compliance cannot be that large to fully explain the positive findings.

Furthermore, reliability of the GAF at the individual level is not sufficient and this could lead to random misclassification in the propensity scores [44]. Some FACT patients may, therefore, not have been matched to the "closest" control patient. However, because this misclassification is random it only leads to noise and a larger confidence interval, while the effect size remains.

Despite the limitations, it is a unique study that combines the merits of an observational study with a careful statistical procedure, resulting in promising results for FACT in the Netherlands.

## Abbreviations

ACT: Assertive Community Treatment; BPRS: Brief Psychiatric Rating Scale; CNCR: Cumulative Needs for Care Register; FACT: Function-ACT (a Dutch variant on ACT); GAF: Global Assessment of Functioning; MMI: Moderately Mental Ill; PCR: Psychiatric Case Register; SMI: Severe Mental Illness.

## Competing interests

The authors declare that they have no competing interests.

## Authors' contributions

MM performed the analyses and wrote the first version of the paper. MD matched the FACT patients with the non-FACT controls, supervised the analyses and edited the first drafts of the paper and revised the paper after the reviewers' comments. MB contributed during interpretation of the analyses and edited various drafts of the paper because of his expertise as a psychiatrist. GD is responsible for the PCR data collection and he matched CNCR data with PCR data; he edited the final draft of the paper. JaC, AdB and GP implement(ed) the CNCR in their regions/institutions and edited the final draft of the paper. JvO and PhD are scientific coordinators of the CNCR, were involved in the interpretation of the results and edited the paper. The final version has been read and approved by all authors.

## Pre-publication history

The pre-publication history for this paper can be accessed here:


